# Supplementary material for: Boundaries steer the contraction of active gels
Source: Nat Commun. 2016 Oct 14;7:13120. doi: 10.1038/ncomms13120 (PMC5067607; doi:10.1038/ncomms13120)
Supplement: Supplementary Information — Supplementary Figures 1-4, Supplementary Notes 1-5, Supplementary References [file ncomms13120-s1.pdf]

## Supplementary Figures

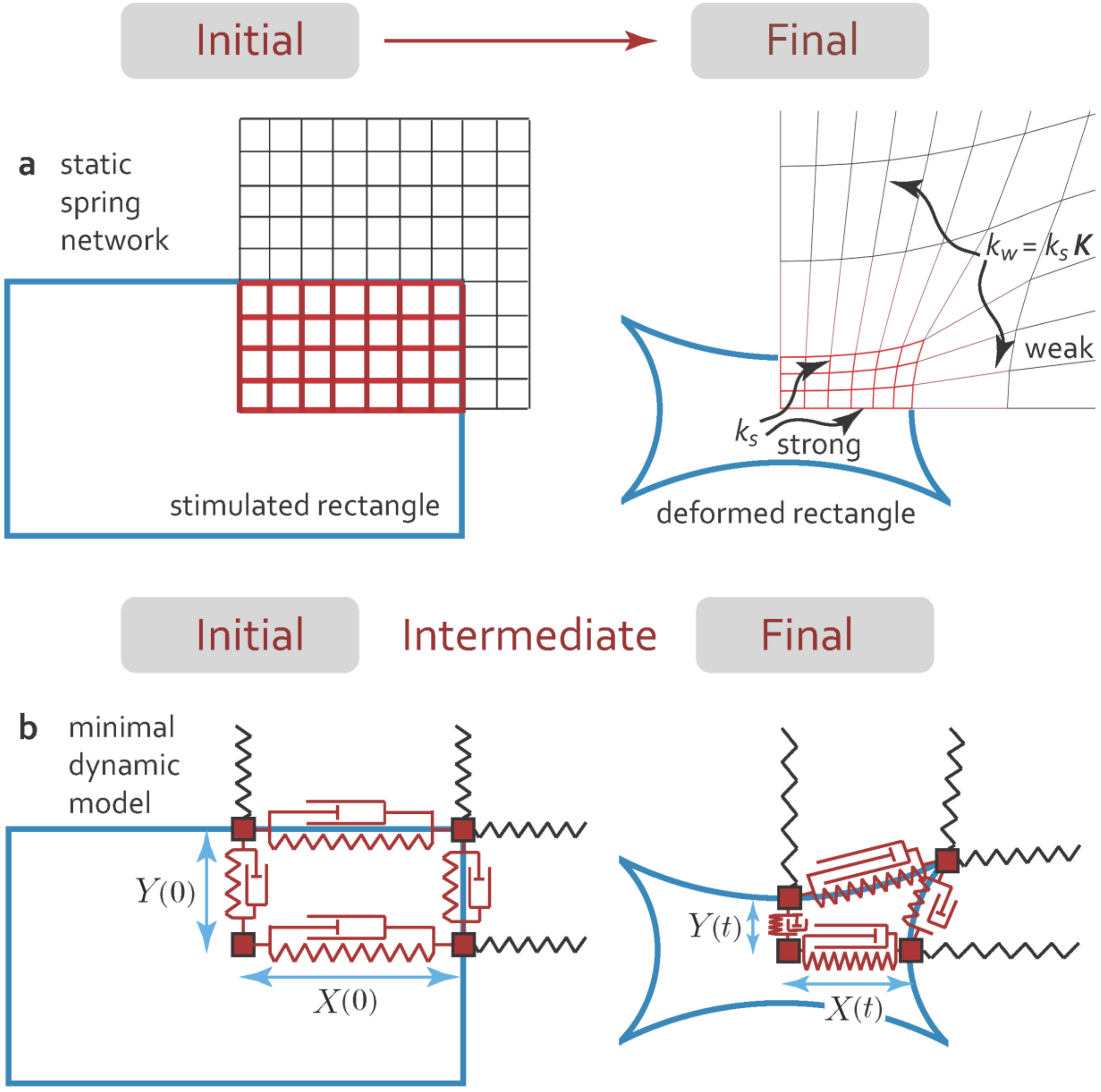


**Supplementary Figure 1**: **Models**. **(a)** Static spring network model, that exhibits an increased stiffness within the stimulated (blue), here rectangular area initially (left), and evolves instantaneously towards its equilibrium state (right) by matrix inversion. **(b)** The minimal dynamical model is defined by a minimal amount of effective springs connecting the symmetry center with characteristic points residing on the interface between active and passive regions. The black squares represent masses, and the dashpots represent friction. The active (strong, red) and passive (weak, black) springs residing within the stimulated area and infinite surrounding, respectively, result in a net spring acting on the mass. The minimal dynamic model contains the spring coefficient for this net spring (equation 3), and a related compression time for each dimension. While the dynamics is governed by the compression time, the static behavior is for both models fully characterized by the same $K$ .

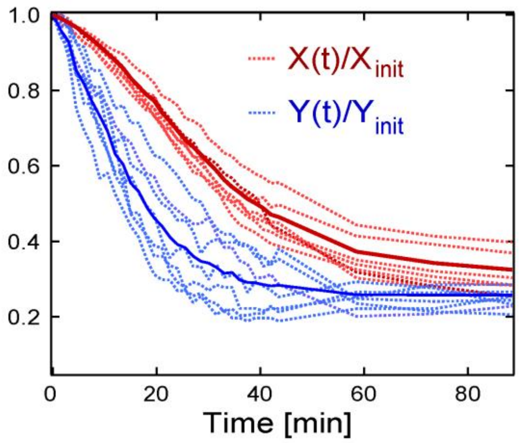


**Supplementary Figure 2: Additional data**. Measured contraction ratios for the lengths $X(t)/X_{\text{init}}$ (red) and widths $Y(t)/Y_{\text{init}}$ (blue) vs. time after stimulation of a series of rectangles with initial axis ratios $2 \le \alpha_{\text{init}} \le 15$ but constant initially stimulated area $X_{\text{init}}Y_{\text{init}} = 10,000$ µm². To guide the eye, thick lines represent a plain average over contraction ratios along the length and the width versus time. All individual data is well captured by equation (3), as demonstrated in Fig. 3a.

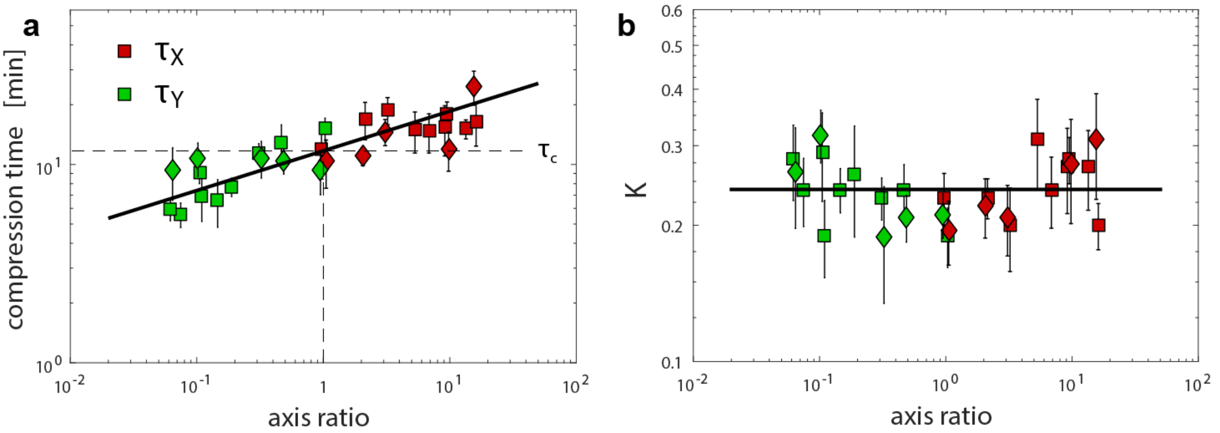


**Supplementary Figure 3: Fitting parameters for full rectangles**. Parameters and their standard deviations are obtained by fitting the measured $X(t)$ and $Y(t)$ to equation (3). **(a)** Contraction times $\tau_X$ (red) and $\tau_Y$ (green) and **(b)** $K$ for the two directions, versus initial axis ratio $\alpha_{\text{init}}$. Black curves show in (a) the model equation (13) for $\gamma = 0.4$ and $\tau_c$ = 12 min, and in (b) a constant $K = 0.23 \pm 0.04$, because $K$ we expect to be unaffected by axis ratio at unchanged stimulation density. Squares and diamonds are used for series of experiments where $X_{\text{init}}$ and the stimulated area had been kept fixed, respectively.

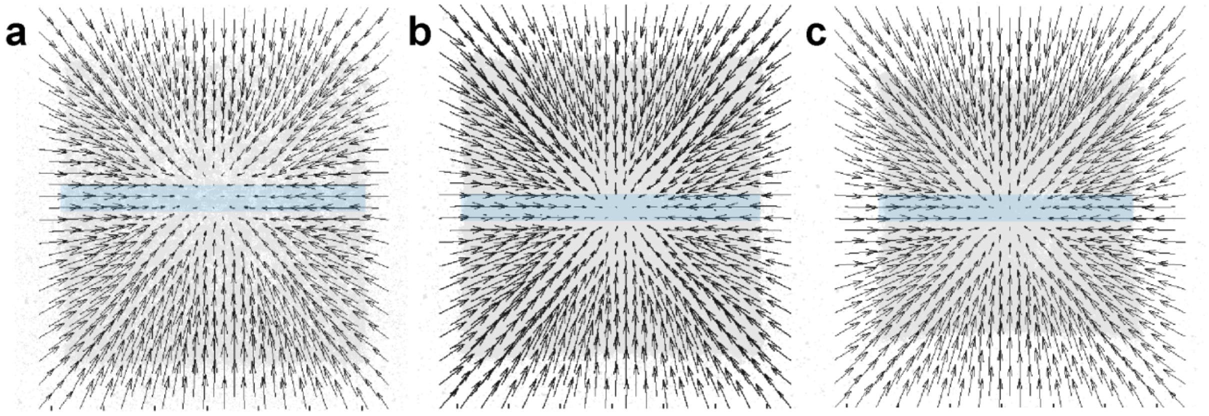


**Supplementary Figure 4: PIV results corresponding to Fig. 2c** at times *t* = **(a)** 5 min, **(b)** 11 min, and **(c)** 17 min. The original PIV contains 4900 displacements (black arrows). Here we show every 8th arrow to distinguish individual ones. To evaluate contraction rates (Figs. 2c,d) deformation gradients along rows are chosen, whose arrows (including those of the neighboring rows) are parallel to these principal axes. All individual spatial gradients for rows are averaged to obtain the profile shown at the respective time point in Fig. 2c. The regions for evaluation are highlighted by light blue horizontal bars and limited by the extent of the active part of the gel, here shown in the background (gray).

# Supplementary Notes

## Supplementary Note 1. Effect of stimulated geometry on final structure

Within our static spring network model the passive, initial, force-free gel is represented by a regular square grid $\mathbf{x}_{\mathrm{init}}$ of crosslinks (nodes) residing within a periodic simulation cell, where each crosslink is permanently connected by four identical harmonic springs to its four nearest neighbors (Supplementary Fig. 1a). While the rest lengths of all individual springs vanish, the initial gel does not tend to shrink to a point but is force-free due to periodic boundary conditions at fixed system area. The system is brought into a highly nonequilibrium state by stimulation: All springs completely residing within the activated area instantaneously increase their spring coefficient by a factor $1/K$. Because we consider harmonic springs, the forces $\mathbf{F}$ are linear in the node positions, $\mathbf{F}(\mathbf{x}) = \mathbf{A} \cdot \mathbf{x} - \mathbf{b}$. The final state is obtained, irrespective any dynamical features that may be added upon assigning masses and friction to the nodes, by requiring mechanical equilibrium, $\mathbf{F}(\mathbf{x}) = \mathbf{0}$, i.e., via $\mathbf{x}_{\mathrm{fin}} = \mathbf{A}^{-1} \cdot \mathbf{b}$. Due to translational invariance, there are exactly two more unknowns than equations that can be used to keep the center of mass of the activated area at its original position. The solution $\mathbf{x}_{\mathrm{fin}}$ is generally system size dependent but converges to a unique result with increasing system size. For a stimulated circle we find that the contraction ratio is identical with $K$, and $K$ is experimentally varied as discussed in Supplementary Notes 2.

This publication contains Supplementary Software, *Mathematica*™ notebook, where we do not setup and invert the matrix $\mathbf{A}$ and vector $\mathbf{b}$, but instead construct $\mathbf{F}$ symbolically. The script essentially solves $\mathbf{F}(\mathbf{x}) = \mathbf{0}$. For large grids, because $\mathbf{A}$ is sparse, it is not recommended to invert $\mathbf{A}$ but instead use a conventional solver adapted to a sparse problem.

## Supplementary Note 2. Multiple stimulations

If we denote with $S_n$ the amount of stimulated motors after n stimulation cycles, $S_\infty$ is the amount of motors that can potentially be stimulated and $S_0 = 0$. The contraction ratio $K = C_{\text{fin}} / C_{\text{init}}$ of a circle with radius $C$ will then depend on n as well and reach $K_\infty$ when there are no motors left to be stimulated. If stimulated motors cannot be stimulated twice, $S_n = S_{n-1} + P(S_\infty - S_{n-1})$ with some stimulation efficiency $P \in [0,1]$. This recursive relationship is solved by $S_n / S_\infty = 1-(1-P)^n$. Because $K$ can be expected to depend linearly on the fraction of stimulated motors, we obtain

$$K = K(n) = 1 + (K_\infty - 1)[1-(1-P)^n] \tag{1}$$

For the characteristic variation of contraction ratio with $n$, from $K = 1$ in the absence of stimulation $(n = 0)$ to $K = K_\infty < 1$ at $n \to \infty$. While $K_\infty$ is an important characteristics of the sample, $P$ is related to the coupling 'strength' between motors and activating light. The relationship (1) is confirmed by Fig. 2a.

## Supplementary Note 3. Minimal dynamic model

Solving the dynamical extension of the spring network model, supplemented by inertia and friction on a large grid would not result in an analytical insight. We instead introduce a minimal dynamic model to capture the full dynamical evolution of the interface between passive and active gel. For the case of a stimulated rectangle the interface can qualitatively be characterized already by the few lengths shown in Supplementary Fig. 1b. The dynamics of these four lengths can be modeled by replacing the large grid of nodes by a minimum amount of only four active, and four passive, critically damped harmonic springs, as shown in Supplementary Fig. 1b. The springs relax in the course of time, and by using the same parameter $K$ as in the static network spring model, the same final lengths are obtained. This is most easily appreciated by considering an idealized one-dimensional situation of the static spring network model, a linear array of initially equally extended springs (extension $b$), where a finite number of $C_{\text{init}} / b$ strong springs (in series) is connected to an infinite amount of $\lim_{H\to\infty}(H - C_{\text{init}})/b$ weak springs in series. The final position $C_{\text{fin}}$ of the interface between weak and strong springs is determined by force balance at the interface

$$\frac{k_s b}{C_{\text{init}}} C_{\text{fin}} = \lim_{H\to\infty} \frac{k_w b}{H - C_{\text{init}}}(H - C_{\text{fin}}) = k_w b \tag{2}$$

where $k_s b / C_{\text{init}}$ is the effective spring coefficient of $C_{\text{init}} / b$ springs in series. While $K$ is defined by $K = k_w / k_s$ within the static spring network model, $K$ is at the same time identical with the ratio of final and initial lengths, $K = C_{\text{fin}} / C_{\text{init}}$, as a result of the above force balance.

**Shape changes: Observations.** Our minimal dynamic model is based on the following three experimental observations (A)–(C):

**(A)** All lengths $L$ including circle radius $C$, length $X$ and width $Y$ of the filled rectangle monotonically decrease in the course of time from their initial values $L_{\text{init}}$ at $t = t_{\text{init}} = 0$ to final values $L_{\text{fin}}$ (Fig. 3a, Supplementary Fig. 2) in a fashion that is reminiscent of the dynamical behavior of a critically damped harmonic oscillator,

$$L(t) = L_{\text{fin}} + (L_{\text{init}} - L_{\text{fin}})(1 + t/\tau_L)e^{-t/\tau_L} \tag{3}$$

where we measure $L_{\text{fin}}$ and the compression time $\tau_L$ for each experiment characterized by $L_{\text{init}}$, in particular. All our data is excellently fitted by this expression (cf. Fig. 3a for a representative example) and there is no simpler expression that does a comparable good job. For symmetry reasons $\tau_X$ and $\tau_Y$ must derive from the same, yet unknown, function $f$

$$\tau_X = \tau_c f(X_{\text{init}} / Y_{\text{init}}), \qquad \tau_Y = \tau_c f(Y_{\text{init}} / X_{\text{init}}) \tag{4, 5}$$

with $f(1) = 1$ and a compression time $\tau_c$ for squares and circles.

**(B)** For all filled shapes (Fig. 3b) at identical stimulation density

$$L_{\text{fin}} / L_{\text{init}} = K \tag{6}$$

with a ratio $0 \leq K \leq 1$ that does not depend on the choice of $L \in \{C, X, Y\}$, but on the amount of stimulation (Supplementary Notes 2).

**(C)** At small times, the inwards speeds $v_L \equiv |\dot{L}|$ of the centers of the two perpendicular sides of a filled rectangle are identical, c.f. inset of Fig. 3b,

$$\lim_{t \to 0} \frac{v_X(t)}{v_Y(t)} = 1 \tag{7}$$

**Implications.** For the interpretation of the time evolution of $L(t)$ it is important noticing that equation (3) is exactly equivalent with the following differential equation for $L(t)$

$$\ddot{L} = -\frac{2}{\tau_L}\dot{L} - \frac{1}{\tau_L^2}(L - L_{\text{fin}}) \tag{8}$$

subject to initial conditions $L(0) = L_{\text{init}}$ and vanishing initial velocities $L(0) = 0$. The last term is an elastic force due to the competing active and passive regions, the only term that survives in the stationary state. The first term on the right hand represents Stokes' friction. We set out to work out the implications of observations (A)–(C). The time-dependent velocity of the interface is given by the derivative of $L(t)$ (equation 3) with respect to time,

$$v_L(t) = \dot{L}(t) = \frac{(1-K)L_{\text{init}}}{\tau_L^2} t e^{-t/\tau_L} \tag{9}$$

and thus approaches zero at large times $t \gg \tau_L$. Let $\alpha(t) = X(t)/Y(t)$ denote the transient axis ratio, and $\alpha_{\text{init}} = \alpha_{\text{fin}}$ its initial and final values, that are identical according to (B). Writing down equation (9) for $L = X$ and $L = Y$, the time-dependent ratio of velocities for the case of a rectangle is

$$\frac{v_X(t)}{v_Y(t)} = \frac{\tau_Y^2(t)}{\tau_X^2(t)} \alpha_{\text{init}} \exp\left(\frac{t}{\tau_Y} - \frac{t}{\tau_X}\right) \tag{10}$$

Because the interface is initially a rest, $\dot{L}(t) = t\ddot{L}(0) + O(t^2)$ with acceleration $\ddot{L}(0)$ at startup. Observation (C) is thus nothing else than reflecting that the accelerations (~ forces) normal to flat surfaces are initially identical. To appreciate the implication of the finding (C) that the ratio of velocities becomes unity in the limit of $t \to 0$, we Taylor-expand equation (10) about $t = 0$

$$\lim_{t\to 0} \frac{v_X(t)}{v_Y(t)} = \frac{\tau_Y^2(t)}{\tau_X^2(t)} \alpha_{\text{init}} \overset{\text{eq S7}}{=} 1 \tag{11}$$

The compression times $\tau_X$ and $\tau_Y$ are therefore interrelated via

$$\frac{\tau_X}{\tau_Y} = \frac{\tau_c f(\alpha_{\text{init}})}{\tau_c f(\alpha_{\text{init}}^{-1})} = \left(\frac{X_{\text{init}}}{Y_{\text{init}}}\right)^{\gamma} = \alpha_{\text{init}}^{\gamma} \tag{12}$$

with exponent $\gamma = 0.5$, while a slightly different exponent $\gamma = 0.4$ is better compatible with our data. The larger the initial axis ratio, the more the times $\tau_X$ and $\tau_Y$ differ. This is simply due to the identical initial forces, but different final lengths of the two axes. There is a certain variety of functions $f$ with $f(1) = 1$ fulfilling condition (12), but the most natural one, $f(\alpha_{\text{init}}) = \alpha_{\text{init}}^{\gamma/2}$ with $\gamma = 0.4$ seems already compatible with our data (Supplementary Fig. 3), and the explicit expressions for times $\tau_X$ and $\tau_Y$ are therefore

$$\tau_X = \tau_c \alpha_{\text{init}}^{\gamma/2}, \qquad \tau_Y = \tau_c \alpha_{\text{init}}^{-\gamma/2} \tag{13}$$

Using equation (12) or (13), the ratio of velocities for the case of a rectangle we can finally write as

$$\frac{v_X(t)}{v_Y(t)} = \exp\left[\left(\alpha_{\text{init}}^{\gamma} - 1\right)\frac{t}{\tau_X}\right] = \exp\left[\left(\alpha_{\text{init}}^{\gamma/2} - \alpha_{\text{init}}^{-\gamma/2}\right)\frac{t}{\tau_c}\right] \tag{14}$$

While the velocities $v_X(t)$ and $v_Y(t)$ both vanish at startup, and both tend to vanish at large times, their ratio increases exponentially with time, and each velocity separately goes through a maximum in the course of time. Since the acceleration is given by $\ddot{L}(t) = (t^{-1} - \tau_L^{-1})\dot{L}(t)$ according to equation (3), the extremal velocity is reached at $t = \tau_L$ and given by

$$v_L^{\max} = v_L(\tau_L) = \frac{(1-K)}{e} \frac{L_{\text{init}}}{\tau_L} \tag{15}$$

This more general case includes the cases of circles and squares, $L = C$, involving the compression time $\tau_c$. A proportionality between $v_c^{\max}$ and initial distance between boundaries $C_{\text{init}}$ is highlighted by Fig. 2b. Equation (15) is furthermore tested by measuring $v_Y^{\max} \sim Y_{\text{init}}$ (Fig. 3d) for the case of a rectangle where $X_{\text{init}}$ was held fixed. The immediate implication is that $\tau_c$ is independent on $C_{\text{init}}$. In turn, $\tau_X$ and $\tau_Y$ are functions of axis ratio alone and insensitive to the overall size or area of the rectangle.

**Implications for data shown in figures**. In the following we comment on the analytical results shown in figures, as some simple rewriting of the above equations is involved. Experimental results for circles and squares (both characterized by $\alpha_{\text{init}} = 1$) are collected in Fig. 2b. The value for $\tau_c$ is contained in Fig. 2b from the slope $v_c^{\max} / 2C_{\text{init}}$ according to equation (15). The compression time is therefore obtained from the slope in Fig. 2b via

$$\tau_c = \frac{(1-K)}{2e \times \text{slope}} \tag{16}$$

With a typical $K$ between 1/6 and 1/4, and slope ≈ 0.2/800 s$^{-1}$ ≈ 2.5 × 10$^{-4}$ s$^{-1}$ from Fig. 2b we find, in agreement with simulation parameters shown in Supplementary Fig. 3, $\tau_c$ ≈ 12 min for this range of $K$. For the case of a filled rectangle, equation (15) furthermore implies

$$\frac{v_X^{\max}}{v_Y^{\max}} = \alpha^{1-\gamma} \tag{17}$$

with the same exponent $\gamma = 0.4$, as demonstrated by Fig. 3c. For $v_X^{\max}$ shown in Fig. 3d, equation (13) predicts $\tau_X \sim Y_{\text{init}}^{-\gamma/2}$ (because $X_{\text{init}}$ was held fixed), once more in apparent agreement with our results. With equation (13) we have reduced the set of model parameters to only two: $\tau_c$ and $K_\infty$. These two parameters allow us to predict the dynamical behavior of arbitrarily sized and shaped rectangles (or circles) as function of the number of stimulations.

Our data in Fig. 2a shows that both $v_c^{\max}$ and $K = C_{\text{fin}} / C_{\text{init}}$ saturate for large number of stimulations $n \to \infty$. In this limit we read off $K_\infty$ ≈ 0.17 and $v_c^{\max}(K_\infty)$ ≈ 1.4 µm/s for this set of experiments. To arrive at the black curve we write down equation (15) for circles twice,

$$v_X^{\max}(K) = \frac{(1-K)}{e} \frac{C_{\text{init}}}{\tau_c(K)} \tag{18}$$

and the same equation where $K$ is replaced by $K_\infty$. Following our above discussion, $\tau_c$ may be proportional to $K$. This yields the black curve in Fig. 2a,

$$v_c^{\max}(n) = \frac{K_\infty v_c^{\max}(K_\infty)}{1-K_\infty} \times \frac{1-K(n)}{K(n)} \tag{19}$$

with the known numerical prefactor, where $K(n)$ (stated in the manuscript) is the red curve. The time-dependent behavior of the axis ratio $\alpha(t)$ follows from observation (A), i.e., equation (3). While the initial axis ratio is identical with the final one, $\alpha(t)$ goes through a maximum $\alpha_{\max}$ (Fig. 3c) at a certain time, as shown in Fig. 3b. The existence of a maximum follows from observations (B)–(C). The

time at which $\alpha_{\max}$ is reached does not coincide with $\tau_X$ or $\tau_Y$ ! Both the time and value for $\alpha_{\max}$ can be obtained by numerically solving $\dot{\alpha}(t)=0$ for $t$, where $\alpha(t)=X(t)/Y(t)$ is given in (A).

For the experimental data shown in Fig. 3d, $X_{\text{init}}$ was held fixed, while $Y_{\text{init}} \leq X_{\text{init}}$ was varied. If we write down equations (15) and (13) for the two axes $X, Y$ and the square case separately, we obtain

$$v_X^{\max} = \frac{v_c^{\max}}{\alpha_{\text{init}}^{\gamma/2}} = \frac{v_c^{\max}}{X_{\text{init}}^{\gamma/2}} Y_{\text{init}}^{\gamma/2}, \qquad v_Y^{\max} = \frac{v_X^{\max}}{\alpha_{\text{init}}^{1-\gamma}} = \frac{v_c^{\max}}{X_{\text{init}}^{1-\gamma/2}} Y_{\text{init}}^{1-\gamma/2} \tag{20}$$

again with $\gamma = 0.4$. While $v_Y^{\max} \sim Y_{\text{init}}^{0.8}$ should thus vary mostly linearly, $v_X^{\max} \sim Y_{\text{init}}^{0.2}$ should depend weakly on $Y_{\text{init}}$ when $Y_{\text{init}}$ approaches $X_{\text{init}}$, and in overall agreement with our data (Fig. 3d). This representation of data eliminates the ratio of spring coefficients $K$, because it is contained in $v_c^{\max}$.

The cross-dependency expressed by equation (13) highlights the fact, how the dynamics of $X$ and $Y$ are coupled. One possibility to interpret the cross-dependency in a two-dimensional setup stems from considering a harmonic spring that exhibits some equilibrium extension, $r_{\text{fin}}$,

$$\mathbf{F} = -k(r - r_{\text{fin}})\frac{\mathbf{r}}{r} \tag{21}$$

with $r = |\mathbf{r}|$. If such a spring is contained in a small volume of our gel that has (already) the ability to contract in y-direction, but (not yet) the ability to contract in x-direction, the force will tend to shorten the spring, while it gets aligned towards the x-direction. At the time the spring will have received the ability to contract in *x*-direction as well, it has already shortened and weakened. This implies a longer relaxation time for the delayed contraction in *x*-direction, qualitatively expressed by equation (13).

**To summarize**, our observations (A)–(C) lead to predictions for the extension of the stimulated region that were highlighted further in Fig. 2b and Fig. 3a–d. The material characteristics and experimental conditions are contained in the factor of proportionality $\tau_c$ in equation (13) between compression time and $K$. The latter we express in terms of number of stimulations, stimulation efficiency, and limiting contraction ratio in Supplementary Notes 2.

## Supplementary Note 4: Additional experimental data and procedures

Selected PIV results are shown in Supplementary Fig. 4. The time dependent positions of the interface $L(t)$ for full circles (where $L = C$ stands for radius) and rectangles (where $L$ stands for the extension in $X$ or $Y$ direction, or also for their medians for the purpose of Fig. 2b) had been obtained from experimental data via image analysis as described in the Methods section. Within the static spring network calculations (Fig. 1c) the positions $L(t)$ correspond to the positions of the interfacial nodes at $y = 0$ (circle and square). For the minimal dynamic model $L(t)$ is known analytically. In each case the velocity of the interface $\dot{L}(t)$ and in particular also the quantities $v_L^{\text{init}}$ and $v_L^{\max}$ derive from $L(t)$. While the direct evaluation of local slopes of $L(t)$ from experimental data is prone to large error bars, we first fitted the measured $L(t)$ by equation (3) upon assuming that there is an arbitrary

coefficient in front of the $t/\tau_L$ term in the second bracket. This corresponds to the more general case of non-critical damping, and excellently captures our experimental data with a coefficient close to unity. The analytical expression (3) exhibits a maximum that is analytically expressed in terms of the fitting parameters $K$ and $\tau_L$ in equation (15). In so far we do calculate $v_L^{\max}$ and also the extrapolated final extension $L_{\text{fin}}$ with a small error from the available $L(t)$ curve. The compression time $\tau_L$ does not affect stationary results, because the force balance is unaffected by inertia and friction.

For the stationary results, the only fitting parameter is $K$. If one determines the time $\tau_L$ for which the velocity goes through an extremum, and the final extension $L_{\text{fin}}$, all experimental data falls onto a single curve if $(L(t)-L_{\text{fin}})/(L_{\text{init}}-L_{\text{fin}})$ is plotted against $t/\tau_L$, as is evident from equation (3).

## Supplementary Note 5: Related phenomena

We are aware of a single nontrivial case where a closely related problem had been studied analytically. Eshelby[1] investigated the case of an ellipsoidal elastic inclusion in an infinite elastic body and found an analytic solution solving the continuum equations (limit of vanishing mesh size). The ellipsoid remains an ellipsoid and changes only its axis ratio, which simplifies the problem compared with our filled or hollow rectangles (Fig. 1).

The question on why or under which circumstances critical damping occurs in networks is challenging. Paley *et al.*[2] studied critical damping in a kinetic interaction network theoretically. Such a network transmits information about external cues quickly and accurately. Analysis of a one-dimensional interaction network revealed a bound on the algebraic connectivity above which the transient response is overdamped. A critically damped response, the fastest and most accurate, is achieved by maximizing the algebraic connectivity subject to this bound. Empirical studies of bird flocks, fish schools, or human crowd suggest that topological distance, as opposed to metric distance, may determine the interaction network, cf. Ballerini *et al.*[3,4] A remarkable property of a natural interaction network is the inherent capability to accurately and rapidly transmit information about internal or external cues (stimulation) that are sensed by only a few individuals (at the active-passive interface).

# Supplementary References

[1] Eshelby, J. The determination of the elastic field of an ellipsoidal inclusion and related problems. *Proc. R. Soc. Amer*. **241**, 376--396 (1957).

[2] Paley, D. & Baharani, A. Critical damping in a kinetic interaction network. *Proc. Amer. Control Conf*. **2010**, 4628–4633 (2010).

[3] Ballerini, M., Cabibbo, N., Candelier, R., Cavagna, A., Cisbani, E., Giardina, I., Orlandi, A., Parisi, G., Procaccini, A. & Viale, M. Interaction ruling animal collective behavior depends on topological rather than metric distance: Evidence from a field study. *Proc. Natl. Acad. Sci. USA* **105**, 1232–1237 (2008).

[4] Ballerini, M., Cabibbo, N., Candelier, R., Cavagna, A., Cisbani, E., Giardina, I., Orlandi, A., Parisi, G., Procaccini, A. & Viale, M. Empirical investigation of starling flocks: a benchmark study in collective animal behaviour. *Animal Behav*. **76**, 201–215 (2008).
